# Validation of replacement questions for slowness and weakness to assess the Fried Phenotype: a cross-sectional study

**DOI:** 10.1007/s41999-020-00337-8

**Published:** 2020-06-04

**Authors:** Michael C. J. Van der Elst, Birgitte Schoenmakers, Linda P. M. Op het Veld, Ellen E. De Roeck, Anne Van der Vorst, Jos M. G. A. Schols, Jan De Lepeleire, Gertrudis I. J. M. Kempen, D-SCOPE Consortium

**Affiliations:** 1 Department of Public Health and Primary Care, University of Leuven, Kapucijnenvoer 33 Bus 7001, 3000 Leuven, Belgium; 2 Department of Health Services Research, Care and Public Health Research Institute (CAPHRI), Maastricht University, P.O. Box 616, 6200 MD Maastricht, The Netherlands; 3 Faculty of Health, Centre of Research Autonomy and Participation for Persons with a Chronic Illness, Zuyd University of Applied Sciences, Heerlen, The Netherlands; 4 Clinical and Lifespan Psychology, Vrije Universiteit Brussel, Brussel, Belgium; 5 Laboratory of Neurochemistry and Behavior, University of Antwerp, Antwerp, Belgium; 6 Department of Family Medicine, Faculty of Health, Medicine and Life Science, Maastricht University, Maastricht, The Netherlands

**Keywords:** Frailty, Fried Phenotype, Screening, Performance-based test, Self-report question

## Abstract

**Purpose:**

When screening large populations, performance-based measures can be difficult to conduct because they are time consuming and costly, and require well-trained assessors. The aim of the present study is to validate a set of questions replacing the performance-based measures slowness and weakness as part of the Fried frailty phenotype (FRIED-P).

**Methods:**

A cross-sectional study was conducted among community-dwelling older adults (≥ 60 years) in three Flemish municipalities. The Fried Phenotype (FRIED-P) was used to measure physical frailty. The two performance-based measures of the Fried Phenotype (slowness and weakness) were also measured by means of six substituting questions (FRIED-Q). These questions were validated through sensitivity, specificity, Cohen’s kappa value, observed agreement, correlation analysis, and the area under the curve (AUC, ROC curve).

**Results:**

196 older adults participated. According to the FRIED-P, 19.5% of them were frail, 56.9% were pre-frail and 23.6% were non-frail. For slowness, the observed sensitivity was 47.0%, the specificity was 96.5% and the AUC was 0.717. For weakness, the sensitivity was 46.2%, the specificity was 83.7%, and the AUC was 0.649. The overall Spearman correlation between the FRIED-P and the FRIED-Q was *r* = 0.721 with an observed agreement of 76.6% (weighted linear kappa value = 0.663, quadratic kappa value = 0.738).

**Conclusions:**

The concordance between the FRIED-P and FRIED-Q was substantial, characterized by a very high specificity, but a moderate sensitivity. This alternative operationalization of the Fried Phenotype—i.e., including six replacement questions instead of two performance-based tests—can be considered to apply as screening tool to screen physical frailty in large populations.

**Electronic supplementary material:**

The online version of this article (10.1007/s41999-020-00337-8) contains supplementary material, which is available to authorized users.

## Key summary points

**Aim:**

What is the overall concordance between FRIED-P and FRIED-Q?

**Findings:**

The concordance between the FRIED-P and FRIED-Q was substantial, characterized by a very high specificity but a moderate sensitivity.

**Message:**

The FRIED-Q can be used as a step in a sequential process to detect frailty in a large population.

**Electronic supplementary material:**

The online version of this article (10.1007/s41999-020-00337-8) contains supplementary material, which is available to authorized users.

## Introduction

Physical frailty is a state of increased vulnerability, which can evolve into disability and other adverse outcomes [1–5]. However, frailty in older adults is often not identified [3, 6, 7]. Large-scale screening may be helpful to identify frail older persons [8]. However, to implement large-scale screening, an easy to apply frailty screener is necessary [3]. One of the most frequently used scales to assess frailty is the Fried Phenotype [9, 10]. According to the Fried Phenotype, a person is frail when he or she meets at least three of the following criteria: unintentional weight loss, slowness, weakness, exhaustion, and low physical activity [11]. Slowness and weakness are both assessed with performance-based measures. When large populations of older people are screened, performance-based measures can be difficult to conduct because they are time consuming, costly, and require well-trained assessors [12]. Consequently, the replacement of these performance-based measures by self-report questions may be helpful in the development of an easy to apply frailty-screening tool, which may enable to screen large populations [6, 10]. Although the two performance-based criteria (slowness and weakness) of the Frailty Phenotype were already replaced by questions in a few earlier studies (e.g., Santos-Eggimann and colleagues 2009, Gordon and colleagues 2020) [13, 14], still little is known about which questions (or set of questions) are most valid to substitute the performance-based measures [10]. In most of the studies in which self-report questions were used, the validity of these questions was not tested, or at least not reported, while these modifications may have an important impact on its classification and predictive ability [10, 15].

Nonetheless, a recent study did test the psychometric properties of six self-report questions to replace the performance-based measurements slowness and weakness [15]. In this study by Op het Veld and colleagues [15], participants were recruited from different settings in the Netherlands: a community center for older people, clients of a physical therapy practice, people admitted to a hospital, and people attending day care facilities. It was aimed to include 50 persons per frailty stage (i.e. frail, pre-frail, non-frail). Regarding the psychometric properties, this study showed an observed agreement of 71.1% between a Fried Phenotype with performance-based measures and a Fried Phenotype without performance-based measures, but including self-report questions and a Cohen’s kappa = 0.55 [11, 15].

Whereby the study of Op het Veld and colleagues was explorative, the aim of the present study is to validate and to confirm the psychometric properties of this set of six self-report questions [15]. However, some differences in setting between both studies occur. The present validation study was done in a Flemish sample, while Op het Veld and colleagues did their study in the Netherlands, whereby the present sample was larger. While the study of Op het Veld and colleagues was organized in several settings (e.g., a community center for older people, clients of a physical therapy practice, people admitted to a hospital, and people attending day care facilities) whereby the older adults visit the care provider, the present study must be placed in the context of the D-SCOPE framework which aims to detect frail older adults proactively (care providers visiting older adults). The recruitment in the present study is based on census records (and risk factors) and without aims with regard to frailty stages of the sample.

The research questions of the present study are: (1) What is the concordance between slowness operationalized by doing a 15 ft. walk time test and slowness operationalized by four self-report questions? (2) What is the concordance between weakness measured by means of a handgrip strength test and weakness operationalized by two self-report questions? (3) What is the concordance between the two overall operationalization’s of the Fried scales? (4) What is the ability of the Fried Phenotype with no performance-based tests to discriminate between non-frail and/or frail older adults if we take the Fried Phenotype with performance-based test as a gold standard?

## Method

### Study design

For this cross-sectional study, data were gathered as baseline wave within the D-SCOPE project [16]. D-SCOPE stands for Detection, Support and Care for Older adults: Prevention and Empowerment. The aim of D-SCOPE was to detect frail community-dwelling older adults who previously were unnoticed and to improve their access to tailored care and support. The details of the data collection method of D-SCOPE have been published elsewhere [16]. To determine the numbers of participants needed, a sample size calculation was conducted a priori (see Online Resource 1: Sample size) [17]. This resulted in a required minimum of 138 participants to be able to show a statistically significant effect (*p* < 0.05) by means of a correlation of 0.30. Participants had to be community dwelling and 60 years or older and were selected from the census records, based on risk profiles (e.g., age, gender, marital status, country of birth) developed by Dury and colleagues [16, 18]. Participants were excluded from the study in case of hospitalization, when inability to participate was indicated by the participant or his/her informal caregiver, or when the interviewer noted that the older participant was unable to provide adequate answers (e.g., not being able to answer questions due to physical exhaustion or distraction). The present study took place in three Flemish municipalities Ghent, Knokke-Heist and Thienen in Belgium. To minimize intra- and inter-assessor variability, the collection of the data was performed by two trained interviewers (authors MCJVdE and AVdV). Data collection started in March 2017 and ended in September 2017. This study was reviewed and approved by the Medical Ethics Committee of the Vrije Universiteit Brussel, Brussels, Belgium (reference number: B.U.N. 143,201,630,458). Written consent was obtained from all participants. The study adheres to the STROBE guidelines.

### Frailty measurements

Fried’s Phenotype of physical frailty was used to measure frailty [11]. The Fried Phenotype uses five criteria to determine the level of frailty: weight loss, exhaustion, low physical activity, slowness, and weakness [11]. Slowness and weakness were measured both in a performance-based way as proposed by Fried and colleagues [11], and additionally by using the six replacement questions as proposed by Op het Veld and colleagues (see Online Resource 2: development of the replacement questions) [15]. A detailed description of the performance-based measurements and its cutoffs are given in Online Resource 3: frailty measurement [11, 15]. Each frailty criterion was recoded in a dichotomous score: frail (score 1) or non-frail (score 0). The final frailty sum scores range from 0 to 5 and classify persons into non-frail (score 0), pre-frail (score 1–2) or frail (score 3–5). In what follows, the Fried Phenotype with performance-based measures is named FRIED-P, and the Fried Phenotype replacing the performance-based measures by six questions is named FRIED-Q. Table 1 presents an overview of the criteria and descriptions for both FRIED-P and FRIED-Q.

**Table 1:** Fried Phenotype: FRIED-P including performance-based measures for weakness and slowness and FRIED-Q including self-report questions for weakness and slowness

	FRIED-P	FRIED-Q
Weight loss	In the last year, have you lost more than 10 lb unintentionally?	In the last year, have you lost more than 10 lb unintentionally?
Exhaustion	How often in the last week did you feel this way? (a) I felt that everything I did was an effort (b) I could not get going	How often in the last week did you feel this way? (a) I felt that everything I did was an effort (b) I could not get going
Low physical activity	Do you do sports activities (e.g., walking, swimming, or cycling)?	Do you do sports activities (e.g., walking, swimming, or cycling)?
Weakness	Participants were asked to squeeze as hard as possible on the dynamometer (Saehan)	(1) Do you have trouble watering plants with a spray bottle? (2) Do you feel like you have less hand strength than other people your age?
Slowness	Participants were asked to walk time/15 feet	(1) When the doorbell rings, do you usually get there in time to open the door? (2) Do you walk more slowly than you would like? (3) Do you have enough time to cross the street on foot when the traffic light turns green? (4) Do you encounter problems in daily life due to poor balance?

An item was positive if: (a) weight loss was answered with yes; (b) exhaustion was answered with 3–4 days or more a week to either of these questions; (c) low physical activity was answered monthly or less; (d) weakness was answered yes on at least one question; and (e) slowness had a score 3 or higher. For slowness every question was assigned a score 1, except question 2 which was assigned a score of 2, since it contributed substantially more to the total score than any of the other questions. The scores were summed (0–5), and the cutoff score is 3

### Statistical analyses

To describe the population, univariate descriptive statistics were conducted. To get an impression whether the items of slowness and the items weakness are related, the mean inter-item correlations were calculated for both measurements. A low inter-item correlation suggests that the items are hardly related to each other and might not be suitable for measuring a single construct. A high inter-item correlation suggests that the items tend to be very similar to each other, almost to the point that they are redundant. Optimal mean inter-item correlation values range from 0.2 to 0.4 [19].

In research questions 1 and 2, we examine the concordance between the performance-based test (‘gold standard’, hand grip strength and walk time) and the replacement questions, to have a better understanding of the concordance if several tests were applied: sensitivity, specificity, observed agreement, Cohen’s kappa (interrater reliability); the performance of the model for both hand grip strength and walk time was quantified as the area under the receiver operating characteristic curve (AUC) [20–22].

To measure the AUC, the scores on the replacement question of hand grip strength and walk time were used as test variable and the score on the performance-based test was used as state variable.

To measure the concordance between the FRIED-P and the FRIED-Q (research question 3), the Spearman correlation and observed agreement were computed. Since the Fried Phenotype has three categories, frail, pre-frail and non-frail, a weighted kappa value (linear and quadratic) was calculated, whereby the FRIED-P was used as the ‘gold standard’.

To measure the ability of the FRIED-Q to discriminate between non-frail and/or frail people (research question 4), the sensitivity, specificity, Cohen’s kappa, observed agreement and area under the receiver operating characteristic curve (AUC) were measured against the FRIED-P.

The interpretation of the (Cohen’s) kappa value was divided as follows: < 0: poor; 0–0.20: slight; 0.21–0.40: fair; 0.41–0.60: moderate; 0.61–0.80: substantial; 0.81–1.00: almost perfect [23]. The area under the curve (AUC, ROC curve) was interpreted as follows: 90–100 = excellent; 80–90 = good; 70–80 = fair; 60–70 = poor; 50–60 = fail [24]. Cases with missing data were excluded pairwise. The statistical analyses were performed using SPSS 24 (IBM SPSS Statistics for Windows, IBM Corp., Armonk, NY, USA).

## Results

In total, 196 participants participated in the study with an average age of 72.7 (SD 8.0) of which 49.0% was male. The characteristics of the population are further described in Table 2.

**Table 2:** Descriptive statistics of the study sample (*N* = 196)

	Mean (SD)	*N* (%)
Age	72.7 (8.0)	
Range age	60–95	
Gender		
Male		96 (49.0)
Female		100 (51.0)
Marital status		
Married		61 (31.1)
Never married		14 (7.1)
Divorced		42 (21.4)
Cohabited		26 (13.3)
Widow (ed)		53 (27.0)
Education		
No/primary		8 (3.1)
Lower secondary		58 (29.7)
Higher secondary		77 (39.5)
Higher education		52 (26.7)
Relocated past 10 years		
Yes		97 (49.5)
No		99 (50.5)
Origin		
Flemish		176 (89.8)
Other		20 (10.2)

According to the FRIED-P, 19.5% of the population was frail, 56.9% was pre-frail, and 23.6% was non-frail (not tabulated). According to the FRIED-Q 14.6% was frail, 52.1% was pre-frail, and 33.3% was non-frail (not tabulated). For the four questions related to slowness, the mean inter-item correlation was 0.266, which is between the range of the optimal inter-item correlation. The mean inter-item correlation value for weakness was 0.221, which is also between the range of the optimal inter-item correlation.

The AUC for slowness was 0.717, which can be defined as fair. The replacement questions for slowness had a sensitivity of 47.0% and a specificity of 96.5% (see Table 3). The observed agreement was 75.5%. The Cohen’s kappa value was *κ* = 0.464, and was defined as moderate.

**Table 3:** The psychometric properties of slowness for the FRIED-Q compared to the FRIED-P

	Performance-based test 15ft Walk time		Total	Positive predictive value	Negative predictive value
	+	−			
Replacement questions					
+					
N	39	4	43		
%	47.0	3.5	21.9	90.7	
Slowness					
−					
*N*	44	109	153		
%	53.0	96.5	78.1		71.2
Total					
*N*	83	113	196		
Sensitivity					
%	47.0				
Specificity					
%		96.5			

FRIED-P stands for the Fried Phenotype with slowness and weakness operationalized as performance-based tests. FRIED-Q stands for the Fried Phenotype with slowness and weakness operationalized as self-report questions. + indicates a participant’s slowness was higher than the cutoff determined by Fried Phenotype or had a higher score than the cutoff on the replacement questions determined by Op het Veld and colleagues (see Online Resource 2), − indicates a participant's slowness was lower than the cutoff determined by Fried Phenotype or had a lower score than the cutoff on the replacement questions determined by Op het Veld and colleagues

The AUC for weakness was 0.649, which can be defined as poor. The replacement questions for weakness had a sensitivity of 46.2% and a specificity of 83.7% (see Table 4). The observed agreement was 73.6%. The Cohen’s kappa value was *κ* = 0.308 and thus defined as fair.

**Table 4:** The psychometric properties of weakness for the FRIED-Q compared to the FRIED-P

	Performance-based test dynamometer		Total	Positive predictive value	Negative predictive value
	+	−			
Replacement questions					
+					
* N*	24	23	47		
%	46.2	16.3	24.4	51.1	
Weakness					
−					
*N*	28	118	146		
%	53.8	83.7	75.6		80.8
Total					
*N*	52	141	193		
Sensitivity					
%	46.2				
Specificity					
%		83.7			

FRIED-P stands for the Fried Phenotype with slowness and weakness operationalized as performance-based tests. FRIED-Q stands for the Fried Phenotype with slowness and weakness operationalized as self-report questions. + indicates a participant’s weakness was lower than the cutoff determined by the Fried Phenotype or had a higher score than the cutoff on the replacement questions determined by Op het Veld and colleagues (see Online Resource 2), − indicates a participant's weakness was higher than the cutoff determined by the Fried Phenotype or had a lower score than the cutoff on the replacement questions determined by Op het Veld and colleagues

The observed agreement of the three frailty stages between FRIED-P and FRIED-Q was 76.6%. The kappa value was substantial (unweighted *κ* = 0.607, weighted linear *κ* = 0.663, weighted quadratic *κ* = 0.738). The Spearman correlation between the FRIED-P and FRIED-Q (5 items) was *r* = 0.721.

When distinguishing between frail and non-frail/pre-frail older adults, the FRIED-Q had a sensitivity of 64.9% and a specificity of 97.4% against the FRIED-P (Table 5). The observed agreement was 91.1% and the area under the curve = 0.811 (ROC) was good. The Cohen’s kappa value was substantial (*κ* = 0.686).

**Table 5:** Ability of FRIED-Q to discriminate between frail and non-frail older adults as compared to the FRIED-P

	FRIED-P		Total	Positive predictive value	Negative predictive value
	Frail	Non-/pre-frail			
Ability of FRIED-Q to discriminate between frail and pre-frail/non-frail					
FRIED-Q					
Frail					
*N*	24	4	28		
%	64.9	2.6	14.6	85.7	
Non-/pre-frail					
*N*	13	151	164		
%	35.1	97.4	85.4		92.1
Total					
*N*	37	155	192		
Sensitivity					
%	64.9				
Specificity					
%		97.4			

	FRIED-P		Total	Positive predictive value	Negative predictive value
	(Pre-)Frail	Non-frail			
Ability of FRIED-Q to discriminate between non-frail and pre-frail/frail					
FRIED-Q					
(Pre-)Frail					
N	123	5	128		
%	84.2	10.9	66.7	96.1	
Non-frail					
*N*	23	41	64		
%	15.8	89.1	33.3		64.1
Total					
*N*	146	46	192		
Sensitivity					
%	84.2				
Specificity					
%		89.1			

FRIED-P stands for the Fried Phenotype with slowness and weakness operationalized as performance-based tests. FRIED-Q stands for the Fried Phenotype with slowness and weakness operationalized as self-report questions

When distinguishing between non-frail and frail/pre-frail older adults, the FRIED-Q had a sensitivity of 84.2% and a specificity of 89.1% against FRIED-P (see also Table 5). The observed agreement was 85.5% and the area under the curve = 0.867 (ROC) was good. The Cohen’s kappa value was substantial (*κ* = 0.647).

## Discussion

In the present study, the psychometric properties of a set of six questions replacing the performance-based measures for slowness and weakness as part of the FRIED Phenotype were validated. The concordance between FRIED-P (including performance-based measures for slowness and weakness) and FRIED-Q (including self-report questions for slowness and weakness) was substantial. The FRIED-Q is very well in discriminating physically non-frail older adults (specificity 89.1%), but somewhat less in discriminating frail older adults (sensitivity 64.9%). At an item level, slowness and weakness are characterized by a low sensitivity (47.0% and 46.2%, respectively), but high specificity (96.5% and 83.7%, respectively).

The observed agreement (76.6% versus 71.1%) and Cohen’s kappa value (0.607 versus 0.55) of the total scales (research question 3) are slightly better in comparison with the results of Op het Veld et al. [15]. However, the current study found (slowness and weakness) higher specificity (96.5% versus 86.1%, and 83.7% versus 71.9%, respectively), but lower sensitivity (47.0% versus 69.2% and 46.2% versus 73.2%, respectively) rates at item level. This indicates that the replacement questions have the ability to correctly identify those without physical frailty, whereas their ability to correctly identify those with physical frailty seems to be less adequate compared with the results of the study of Op het Veld and colleagues [15, 20]. A first plausible explanation for the differences may be related to the composition of the sample. In the present study, 19.5% of the population was frail, 56.9% was pre-frail and 23.6% was non-frail, while in the study of Op het Veld and colleagues much less people were pre-frail (40.7%) and much more people were non-frail (38.5%) [15]. A second explanation could be related to the way participants were recruited. In the present study, older adults were selected from the census records based on risk factors, while in the study of Op het Veld and colleagues older adults were recruited from different settings such as clients of a physical therapy practice, people admitted to a hospital, and people attending daycare facilities [15]. A previous study, for example, showed that self-reported levels of disability were higher after the completion of performance-based tests [25]. One can assume that participants undergoing physical therapy will experience physical limitations in real time and be aware of it. This may have influenced their perceptions of their level of daily functioning.

The concordance between the Fried Phenotype performance-based measures (slowness and weakness) and the set of replacement questions at item level is fair. In previous studies, discrepancies between self-report measures and performance-based tests were found. For instance, the results in a prior systematic review indicated a correlation range between 0.60 and 0.86 when the same construct was measured in two different ways [26]. As far as we know, only two other studies reported psychometric properties with regard to the replacement of Fried’s performance-based measures with questions. Johansen and colleagues used the Physical Function scale of the SF-36 as a substitution for the two performance-based measures together and found an overall agreement of 72.5% [27]. In an earlier attempt to operationalize the Fried Phenotype into an easy to apply screening tool (GFST), Cherubini and colleagues reported an observed agreement of 70.64% and a kappa value of 0.45 [6]. However, Cherubini and colleagues added extra items like living alone and memory complaints [6]. Consequently, it is difficult to compare the results of both studies with the present study.

When distinguishing between non-frail older adults and frail older adults, the total FRIED-Q was marked by a high specificity (89.1%), but a rather low sensitivity (64.9%). This indicates that people might overestimate their own physical performance while filling in the FRIED-Q. For instance, 44 participants reported no slowness (FRIED-Q), while according to the walk time test they were. On the other hand, only a small number of people (four participants) underestimated their own walk speed (slowness). Previous research found several confounding factors for overestimating own physical competences, such as perceived physical competence, perceived health status, personal control or mastery, and depressive symptomatology [28–30]. For instance, Ferrer and colleagues describe that a person rating his/her health as poor is more likely to overreport functional limitation, while a person that perceives his/her health as good tends to underreport functional limitations. Consequently, one can assume that the present sample perceived their health as good or had a high level of mastery. However, this was not assessed, since this was not the aim of the current study.

In the present study, the prevalence is higher than in comparison with previous research. There are several plausible reasons which can explain why the prevalence of frailty is higher: (1) in the present study, older adults were selected from the census records based on risk factors for frailty. Therefore, the prevalence of frailty will be higher and not representative for the population; (2) differences in inclusion and exclusion criteria, for instance, in the SHARE survey, the sample was aged 50 years and over, while in the D-SCOPE project people had to be 60 years or older [31]; (3) a previous systematic review of Theou et al. showed that modifications in the Fried Phenotype can have an impact on the prevalence of frailty. Since also low physical activity is modified in the present study, this could have an impact on the prevalence of frailty [10].

This study has several strengths and limitations. A strength of the present study is that it replicates the study of Op het Veld and colleagues in a larger sample, whereby it was performed in a different setting and region (Flemish region in Belgium) [15]. Consequently, the present results indicate that this set of questions to replace the performance-based test can be used in different settings/countries. Secondly, the performance-based measurements were carried out under a strict protocol, the same as described in the study of Op het Veld and colleagues [15]. The two assessors in the present study (authors MCJVdE and AVdV) were also trained by Op het Veld. Therefore, we consider that the assessor variability was minimized, which makes a valid comparison between the two studies more likely. A limitation of the present study is the operationalization of the item physical activity, which is different in comparison with the study of Fried and colleagues, and Op het Veld and colleagues [11]. Fried and colleagues used a short version of the Minnesota Leisure Time Activity questionnaire, Op het Veld used adjusted version of the Short Questionnaire to Assess Health-enhancing physical activity (SQUASH), while in the present study we asked ‘Do you do sports activities (e.g., walking, swimming, or cycling)?’ [32, 33]. This difference in operationalization might have affected the observed agreement of the three frailty stages between FRIED-P and FRIED-Q and the kappa value.

The substantial concordance between the FRIED-P and the FRIED-Q suggests the usefulness of the latter to screen frailty in a large population, since the FRIED-Q is easier to apply in comparison with the FRIED-P. The high specificity is an advantage when the objective is to exclude non-frail persons, for instance in (research) projects where being frail is often an inclusion criterion). However, the FRIED-Q does not detect all frail older adults (according the Fried Phenotype) and can be considered as a step in a sequential process to detect frailty in large populations. This sequential process should reduce the number of false positives and false negatives. For instance, most older adults (aged 75 and over) in Europe consult their GP frequently. If large screening of frailty becomes a responsibility of general practitioners, there are frequently occasions to screen the patient. In case of doubt the performance-based tests can still be applied as a second order. Future research is needed to validate these sets of substitution questions in other languages and settings.

## Electronic supplementary material

Below is the link to the electronic supplementary material.

Supplementary file1 (DOCX 30 kb)

Supplementary file2 (DOCX 208 kb)

Supplementary file3 (DOCX 14 kb)

Supplementary file4 (DOCX 20 kb)

## Data Availability

The datasets generated for and analyzed during the current study are not publicly available. Nonetheless, data are available from the corresponding author upon reasonable request.
