# Supplementary material for: Validation of replacement questions for slowness and weakness to assess the Fried Phenotype: a cross-sectional study
Source: Eur Geriatr Med. 2020 Jun 4;11(5):793–801. doi: 10.1007/s41999-020-00337-8 (PMC7550376; doi:10.1007/s41999-020-00337-8)
Supplement: Supplementary file 2 — Supplementary file2 (DOCX 208 kb) [file 41999_2020_337_MOESM2_ESM.docx]

**Supplementary Material 1: Sample size calculation**

Title: Validation of Replacement Questions for Slowness and Weakness to Assess the Fried Phenotype: a Cross-sectional Study.

Journal: European Geriatric Medicine

Name: Michael C.J. Van der Elst MSc,

Affiliation:

-University of Leuven, Department of Public Health and Primary Care, Leuven, Belgium

-Maastricht University, Department of Health Services Research and Department of Family Medicine, Care and Public Health Research Institute (CAPHRI), Maastricht, the Netherlands

E-mail: Michael.vanderelst@kuleuven.be
