# Supplementary material for: Validation of replacement questions for slowness and weakness to assess the Fried Phenotype: a cross-sectional study
Source: Eur Geriatr Med. 2020 Jun 4;11(5):793–801. doi: 10.1007/s41999-020-00337-8 (PMC7550376; doi:10.1007/s41999-020-00337-8)
Supplement: Supplementary file 3 — Supplementary file3 (DOCX 14 kb) [file 41999_2020_337_MOESM3_ESM.docx]

**Supplementary material 2: development of the replacement questions**

Title: Validation of Replacement Questions for Slowness and Weakness to Assess the Fried Phenotype: a Cross-sectional Study.

Journal: European Geriatric Medicine

Name: Michael C.J. Van der Elst MSc,

Affiliation:

-University of Leuven, Department of Public Health and Primary Care, Leuven, Belgium

-Maastricht University, Department of Health Services Research and Department of Family Medicine, Care and Public Health Research Institute (CAPHRI), Maastricht, the Netherlands

E-mail: Michael.vanderelst@kuleuven.be

Questions on walk time and handgrip strength were derived from various sources. First, multiple databases were searched using terms related to frailty, grip strength, and walk time. Only questionnaires in English or Dutch were included. This resulted in 11 questionnaires with potential useful questions, including the Dutch version of the 36-Item Short-Form Health Survey (SF-36) and the Disability Rating Index (Aaronson et al., 1998; Salen, Spangfort, Nygren, & Nordemar, 1994). These questionnaires were screened for questions that were speciﬁcally related to walk time or handgrip strength. In addition, community-dwelling older people and experts (scientists and physical therapists, all working with frail older people) were interviewed. Based on face validity and consultation with the aforementioned experts, ﬁnal sets of 11 questions for walk time and 10 questions for handgrip strength were composed (Table. The response options for all questions were “Yes” or “No”. afterwards, logistic regression analyses with backward stepwise elimination were performed to ﬁnd the optimal set of questions as a substitute for the performance-based measures. The performance of the model was quantiﬁed as the area under the receiver operating characteristic curve (AUC). Bootstrap-validation was then performed to calculate the optimism in the estimation of the AUC.

**reference**

-Op het Veld, L.P., et al., Substitution of Fried’s performance-based physical frailty criteria with self report questions. Archives of gerontology and geriatrics, 2018. **75**: p. 91-95.

| Table: questions |
| --- |
| **Walk time** |
| 1 When the doorbell rings, do you usually get there in time to open the door? |
| 2 When the phone rings, do you usually get there in time to answer it? |
| 3 Do you feel like you walk more slowly than other people your age? |
| 4 Do you walk more slowly than you'd like? |
| 5 When walking with other people your age, do you struggle to keep up? |
| 6 Do other people your age regularly pass you when you're walking? |
| 7 Do you have enough time to cross the street on foot when the traffic light turns green? |
| 8 Do you take approximately two steps per second when walking? |
| 9 Do you encounter problems in daily life due to walking difficulties? |
| 10 Do you encounter problems in daily life due to poor balance? |
| 11 Do you have enough time to cross the street at a pedestrian crossing when the light turns green? |
|  |
| **Handgrip strength** |
| 1 Do you have trouble opening a jar that has already been opened? |
| 2 Do you have trouble opening a jar that has not yet been opened? |
| 3 Do you require assistance and/or a device to open the lid of a jar? |
| 4 Do you have trouble watering plants with a spray bottle? |
| 5 Do you have trouble wringing out a facecloth/dishrag? |
| 6 Do you have trouble opening a drinks bottle or a carton of milk that has not yet been opened? |
| 7 Do you have trouble turning on a tap that has been tightly closed? |
| 8 Do you find it difficult or painful to give arm handshake? |
| 9 Do you encounter problems in daily life due to lack of hand strength? |
| 10 Do you feel like you have less hand strength than other people your age? |
