# Supplementary material for: Validation of replacement questions for slowness and weakness to assess the Fried Phenotype: a cross-sectional study
Source: Eur Geriatr Med. 2020 Jun 4;11(5):793–801. doi: 10.1007/s41999-020-00337-8 (PMC7550376; doi:10.1007/s41999-020-00337-8)
Supplement: Supplementary file 4 — Supplementary file4 (DOCX 20 kb) [file 41999_2020_337_MOESM4_ESM.docx]

**Supplementary material 3: Frailty measurement**

Title: Validation of Replacement Questions for Slowness and Weakness to Assess the Fried Phenotype: a Cross-sectional Study.

Journal: European Geriatric Medicine

Name: Michael C.J. Van der Elst MSc,

Affiliation:

-University of Leuven, Department of Public Health and Primary Care, Leuven, Belgium

-Maastricht University, Department of Health Services Research and Department of Family Medicine, Care and Public Health Research Institute (CAPHRI), Maastricht, the Netherlands

E-mail: Michael.vanderelst@kuleuven.be

- Weight loss was measured by asking: In the last year, have you lost more than 10 pounds unintentionally (i.e., not due to dieting or exercise)? If yes, then frail for weight loss criterion.
- Exhaustion was determined using the following two statements of the CES–D Depression Scale (a) I felt that everything I did was an effort; (b) I could not get going. Respondents were asked “How often did you feel this way in the last week?” Respondents could answer with rarely or none of the time (<1 day; score 0), some or a little of the time (1–2 days; score 1), a moderate amount of the time (3–4 days; score 2), or most of the time (score 3). Subjects answering “2” or “3” to either of these questions were categorized as frail by the exhaustion criterion.
- Low physical activity was measured by asking the participants whether they did sports activities (e.g., walking, swimming, or cycling). The response options were never, rarely, monthly or weekly. Participants answering weekly were categorized as non-frail, the other as frail for the low physical criterion.

Slowness and weakness were assessed in two ways:

1. Performance-based measures for slowness and weakness.

For both performance-based measures, all participants received standardized Instructions:

- For slowness, participants were asked to walk 4.57 m (15 ft.) at a normal pace, starting from a standing position. No encouragement was given by the assessor. A walking aid was permitted if necessary. The test was performed three times, and each time the time (in seconds) they needed was measured. For the analyses, the average time was used.
- Weakness (handgrip strength) was measured using a Saehan hand dynamometer (Saehan Corporation, South Korea). Participants were asked to press as hard as possible on the dynamometer. Three measurements per hand were conducted alternately with a minimum of 30 seconds rest between each attempt. Results were averaged per hand, and the highest average score (e.g., either left or right hand, it did not matter whether this was their ‘dominant hand’), was used for analyses. Before conducting the handgrip strength test, participants were asked to practice. Participants were seated upright in a chair without armrests or standing, the shoulder and forearm in neutral position, and the elbow in 90° flexion. The handle position of the dynamometer was determined in such a way that the intermediate phalanges were on the front side of the handle. Participants were verbally encouraged.

For the cut-off values of the performance-based Fried measures, it is necessary to measure weight and height, in order to calculate the Body Mass Index (BMI). Cut-off values for both performance-based measures as described by Fried and colleagues were used [1]. To measure the weight of the participants both researchers used a scale of the brand OMRON. Participants were weighted twice and the average body weight was calculated and used in the analysis. To measure body length, participants were asked to stand against a wall as straight as possible (without shoes). A book with a hard cover was placed on the participant’s head and a post it was used to mark the participant’s body length. Then, the body length was measured using a ruler. This procedure was done three times, and the average body length was used in the analysis. The assessors were trained to conduct the data collection by author LOhV.

- Slowness, stratified by gender and height (gender-specific cutoff a medium height).

Men

Height ≤ 173 cm 7 seconds

Height > 173 cm 6 seconds

Women

Height ≤ 159 cm 7 seconds

Height > 159 cm 6 seconds

- Weakness, stratified by gender and body mass index (BMI) quartiles:

Men

BMI ≤ 24 ≤ 29

BMI 24.1–26 ≤ 30

BMI 26.1–28 ≤ 30

BMI > 28 ≤ 32

Women

BMI ≤ 23 ≤ 17

BMI 23.1–26 ≤ 17.3

BMI 26.1–29 ≤ 18

BMI > 29 ≤ 21

2) Six replacements questions for slowness and weakness

- To operationalize slowness four questions were used: 1) When the doorbell rings, do you usually get there in time to open the door? 2) Do you walk more slowly than you'd like? 3) Do you have enough time to cross the street on foot when the traffic light turns green? 4) Do you encounter problems in daily life due to poor balance?
- Weakness was assessed using two questions: 1) Do you have trouble watering plants with a spray bottle? 2) Do you feel like you have less hand strength than other people your age?

The response options for all questions were “Yes” or “No”.

Previous research showed that question 2 for slowness (i.e. walk time) and question 1 for weakness (i.e. handgrip strength) contributed substantially more to the total score than any of the other questions [2]. Therefore, they were assigned a score of two, while the other questions were given a score of one. The scores were summed for each participant. For walk time a score 3 or more indicates frailty, for weakness a score of 1 or more indicates frailty.

**References**

1. Fried, L.P., et al., *Frailty in older adults: evidence for a phenotype.* The Journals of Gerontology Series A: Biological Sciences and Medical Sciences, 2001. **56**(3): p. M146-M157.

2. ophetVeld, L.P., et al., *Substitution of Fried’s performance-based physical frailty criteria with self-report questions.* Archives of gerontology and geriatrics, 2018. **75**: p. 91-95.
